# Daytime fluctuations of endurance performance in young soccer players: a randomized cross-over trial

**DOI:** 10.1186/s13104-022-06247-1

**Published:** 2022-11-24

**Authors:** Janis Fiedler, Stefan Altmann, Hamdi Chtourou, Florian A. Engel, Rainer Neumann, Alexander Woll

**Affiliations:** 1 Institute of Sports and Sports Science, Karlsruhe Institute of Technology, Engler-Bunte-Ring 15, 76131 Karlsruhe, Germany; 2 TSG ResearchLab gGmbH, 74939 Zuzenhausen, Germany; 3 Institut Supérieur du Sport et de l’Education Physique de Sfax, Université de Sfax, 3000 Sfax, Tunisie; 4 Activité Physique, Sport et Santé, UR18JS01, Observatoire National du Sport, 1003 Tunis, Tunisie; 5 Institute of Sport Science, Integrative & Experimental Exercise Science & Training, Würzburg University, 97070 Würzburg, Germany; 6 Institute of Movement and Sport, University of Education Karlsruhe, 73133 Karlsruhe, Germany

**Keywords:** Circadian rhythm, Soccer, Aerobic exercise, Endurance, Lactate, Heart rate

## Abstract

**Objectives:**

Fluctuations of physical performance and biological responses during a repetitive daily 24-h cycle are known as circadian rhythms. These circadian rhythms can influence the optimal time of day for endurance performance and related parameters which can be crucial in a variety of sports disciplines. The current study aimed to evaluate the daytime variations in endurance running performance in a 3.000-m field run and endurance running performance, blood lactate levels, and heart rate in an incremental treadmill test in adolescent soccer players.

**Results:**

In this study, 15 adolescent male soccer players (age: 18.0 ± 0.6 years) performed a 3.000-m run and an incremental treadmill test at 7:00–8:00 a.m. and 7:00–8:00 p.m. in a randomized cross-over manner. No significant variations after a Bonferroni correction were evident in endurance running performance, perceived exertion, blood lactate levels, and heart rates between the morning and the evening. Here, the largest effect size was observed for maximal blood lactate concentration (9.15 ± 2.18 mmol/l vs. 10.64 ± 2.30 mmol/l, *p* = .110, ES = 0.67). Therefore, endurance running performance and physiological responses during a field-based 3.000-m run and a laboratory-based test in young male soccer players indicated no evidence for daytime variations.

**Supplementary Information:**

The online version contains supplementary material available at 10.1186/s13104-022-06247-1.

## Introduction

Circadian rhythms describe periodic changes in physiological parameters for an approximately 24-h cycle [1]. They are well established for a range of biological parameters like core body temperature, heart rate (HR), blood pressure, and different hormones [1] and are also present in physical performance and related responses [2, 3]. These circadian rhythms are influenced by other parameters like age, light hours, sleeping pattern, or type of exercise but are overall stable [2]. For coaches and athletes (i.e. soccer players), it might be important to consider circadian rhythms as determinants of exercise capacity as well as performance for the best results in competitions [4]. As endurance running performance is related to overall performance in soccer players, and elite players run about 10 km during one game, this motor fitness parameter is of particular interest [5]. Previous research including soccer players found heterogenic results concerning the presence of daytime variation for endurance performance and related physiological responses like lactate or HR [2, 4, 6–13].

Therefore, this study aimed to examine potential daytime variation (morning *vs*. evening) in *i*) endurance running performance during a 3.000-m field run and an incremental treadmill test; and *ii*) blood lactate concentration and HR during the incremental treadmill test.

According to the literature, we hypothesized that (*i*) endurance running performance during the 3.000-m run and the treadmill test would be higher in the evening than in the morning [7, 12, 14, 15] and (*ii*) that both blood lactate levels and HR during the incremental treadmill test would be different between the morning and evening [4, 16–21].

## Material and Methods

### Participants

Fifteen male soccer players (age = 18.0 ± 0.6 years; height = 178.7 ± 5.3 cm; weight = 71.1 ± 6.6 kg), with a regular training volume of three training sessions per week and one soccer match on the weekend, volunteered to participate in this study.

### Procedures

All 15 participants performed a 3.000-m run and an incremental treadmill test (see [22]) on two occasions at two-day times (one in the morning between 7:00 and 8:00 a.m. as well as one in the evening between 7:00 and 8:00 p.m.). Using a cross-over design, the participants were randomly assigned to two groups. Both groups performed the 3.000-m run first and the incremental treadmill test trials second. However, group 1 performed the first trial for both tests (3.000-m run and incremental treadmill test, respectively) in the morning and the second in the evening, while the timing was switched for group 2. All tests were separated by 36 h.

In a first step, all participants performed the two field-based 3.000-m runs on a 400 m running track. The participants were familiar with the 3.000-m run and were instructed to perform the whole 3.000-m run as fast as possible. Time to completion and ratings of perceived exertion (RPE) [23] were recorded after each trial in order to control for exhaustion criteria [24].

In a second step, all participants performed two laboratory-based incremental treadmill tests on a Woodway treadmill (Woodway GmbH, Weil am Rhein, Germany) with a slope of 1%. Each trial started at a running speed of 6 km/h, increasing by 2 km/h every 3 min. After each 3 min-stage, participants rested for 30-s for collection of capillary blood from the earlobe; two participants provided no consent for blood withdrawal and lactate thresholds were estimated using the Ergonizer Software (Ergonizer, Freiburg, Germany). HR was monitored using a Polar system (Polar Electro Oy, Kempele, Finland) throughout the whole test. Athletes were instructed to complete as many stages as possible, and the test was finished at volitional exhaustion. Blood lactate concentration for each stage was analyzed utilizing Biosen C-Line Sport (EKF-diagnostic GmbH, Barleben, Germany).

### Data analysis

Time to completion and RPE were recorded as parameters for the 3.000-m run. Regarding the incremental treadmill test, the following measurement points were chosen to measure one or multiple of the following parameters: blood lactate concentration, HR, and running speed (see [25]):

- rest: immediately before the beginning of the test in a standing position
- individual aerobic threshold (LT): running velocity at which blood lactate concentration begins to rise above baseline levels
- individual anaerobic threshold (IAT): running velocity at LT + blood lactate concentration of 1.5 mmol/l
- maximal running speed (max): running velocity at the point of volitional exhaustion

The following parameters were included for the incremental treadmill test:

- blood lactate concentration (at rest, LT, IAT, and max)
- HR (at rest, LT, IAT, and max)
- running speed (LT, IAT, and max)

### Statistical analysis

Because of the cross-over study design, the existence of possible sequencing effects was calculated by performing an independent t-test between the sum scores (day 1 + day 2 group 1 vs day 1 + day 2 group 2) for each parameter in addition to a sufficient washout period [26]. All Data are available in the Additional file 1.

Daytime variations in all measured variables were calculated using paired t-tests. To correct for multiple testing, the results were adapted by multiplying the p-value with the number of comparisons of the parameter following the Bonferroni correction [27]. In addition, Cohen’s d effect sizes (ES) were calculated to quantify the magnitude of differences between the morning and evening trials: 0.2 ≤ ES < 0.5 was considered a small effect; 0.5 ≤ ES < 0.8 was considered a moderate effect; ES ≥ 0.8 was considered a large effect [28]. Statistical analyses were performed using SPSS statistical software version 26.0 (SPSS, Inc., Chicago, IL). The level for significance was set a priori to 0.05 after the Bonferroni correction.

## Results

The investigation of potential sequencing effects, analyzed using an independent t-test, showed no significant differences between the two groups.

**Fig. 1:**
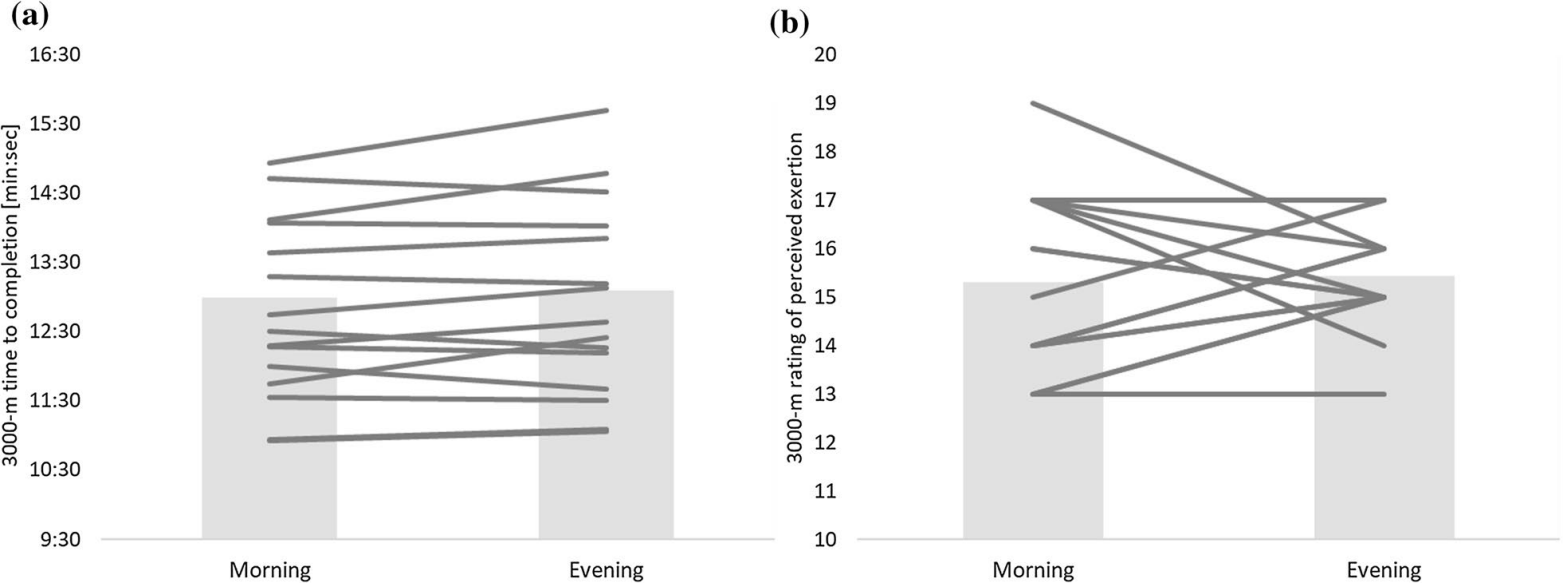
The individual values of all participants (lines) and the mean value (box) for the parameters **a** time to completion, and **b** perceived exertion during the morning and evening trial of the 3.000-m run

**Fig. 2:**
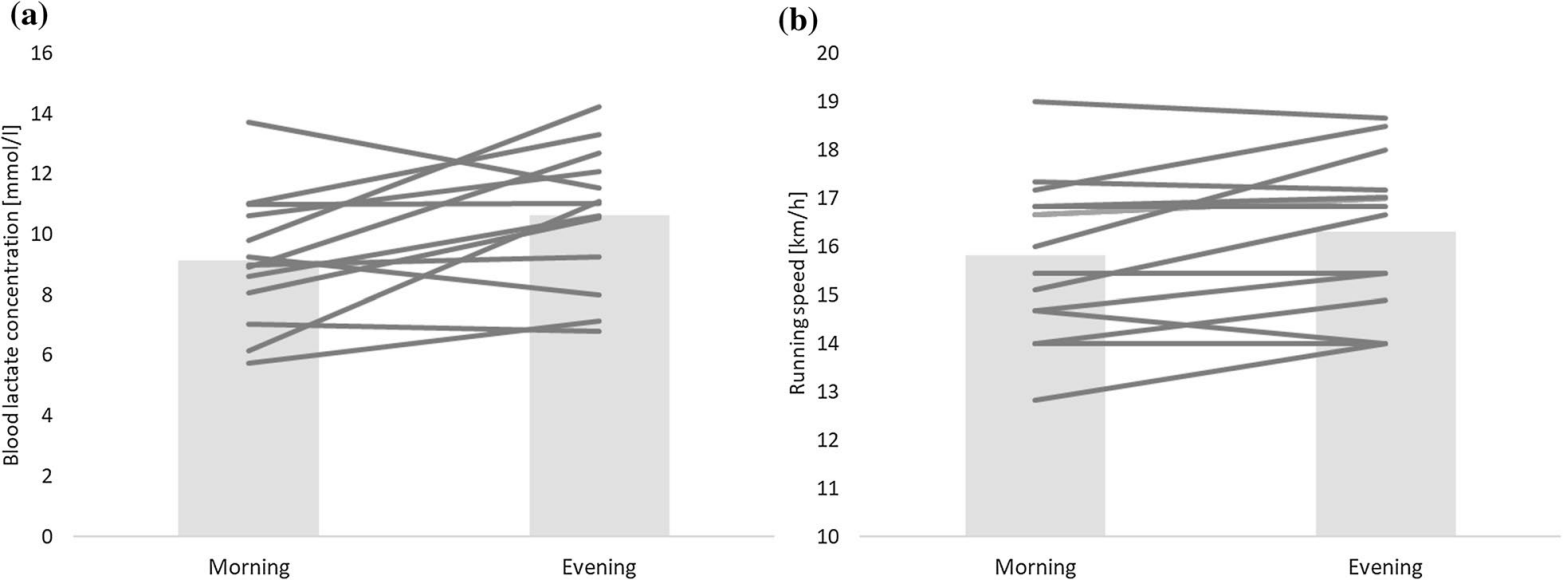
The individual values of all participants (lines) and the mean value (box) for **a** maximal blood lactate concentration, and **b** maximal running speed during the morning and evening trial of the incremental treadmill test

For the 3.000-m run, neither time for completion (see Fig. 1a) nor RPE (see Fig. 1b) differed significantly between the morning and evening trials. For the incremental treadmill test, no significant differences after the Bonferroni correction were found for blood lactate (maximal blood lactate concentration see Fig. 2a) or running speed (maximal running speed see Fig. 2b) between the morning and evening trials (see Table 1 for detailed results).

**Table 1:** Results for endurance running performance, blood lactate levels, and heart rate differences between morning and evening

Incremental treadmill test						
Parameter	Morning	Evening	Mean difference	corrected p-value (original)	Cohen’s d (t-value)	df
Heart rate [1/min]						
Rest	86.60 (9.68)	85.73 (10.88)	0.87 (10.33)	1.00 (0.750)	− 0.09 (0.35)	14
LT	150.93 (12.13)	153.47 (10.29)	− 2.53 (9.81)	1.00 (0.334)	0.23 (− 1)	14
IAT	177.47 (7.85)	179.27 (6.31)	− 1.80 (4.87)	0.872 (0.174)	0.25 (− 1.43)	14
Max	197.13 (6.29)	198.73 (6.08)	− 1.60 (4.21)	0.814 (0.163)	0.26 (− 1.47)	14
Lactate concentration [mmol/l]						
Rest	0.84 (0.21)	0.83 (0.31)	0.01 (0.31)	1.00 (0.930)	− 0.04 (0.09)	12
LT	1.52 (0.67)	1.66 (0.61)	− 0.13 (0.40)	1.00 (0.250)	0.22 (− 1.20)	12
IAT	3.02 (0.67)	3.16 (0.61)	− 0.14 (0.40)	0.962 (0.241)	0.22 (− 2.51)	12
Max	9.15 (2.18)	10.64 (2.30)	− 1.49 (2.15)	0.110 (0.028)	0.67 (− 2.51)	12
Running speed [km/h]						
LT	8.67 (1.17)	9.00 (1.10)	− 3.30 (0.74)	0.429 (0.107)	0.29 (− 1.72)	14
IAT	11.94 (1.28)	12.12 (1.18)	− 0.19 (0.68)	1.00 (0.317)	0.15 (− 1.04)	13
Max	15.81 (1.62)	16.31 (1.59)	− 0.49 (0.76)	0.100 (0.025)	0.31 (− 2.51)	14
3.000-m test						
Time [min:sec]	12:59:00 (1:29)	13:06:00 (1:30)	− 0:07 (0:22)	0.228	0.08 (− 1.26)	15
RPE	15.31 (1.82)	15.44 (1.09)	− 0.13 (1.78)	0.783	0.09 (− 0.28)	15

Means (standard deviations) and results of the paired t-tests for daytime differences at the incremental treadmill test before the start (rest), at the onset of lactate accumulation (LT), the individual anaerobic threshold (IAT), and immediately after volitional exhaustion (max) and at the end of the 3.000-m run for time to completion (Time) and rating of perceived exertion (RPE). p-values were corrected using the Bonferroni method

## Discussion and conclusion

This study aimed to evaluate daytime variation in aerobic endurance performance in a 3.000-m run and an incremental treadmill test in young soccer players. Additionally, blood lactate concentrations and HR during the incremental treadmill test were analyzed for daytime differences. Hypothesis (*i)* that aerobic endurance performance would be better in the evening than in the morning could not be verified for the 3.000-m run and the incremental treadmill test. Hypothesis (*ii)* that blood lactate levels and HR during exercise would be higher in the evening could also not be verified.

Aerobic endurance performance in the incremental treadmill test indicated no evidence for differences between the evening and the morning. This is in line with some previous studies in untrained participants [20] and competitive cyclists [29] while others reported increased endurance performance in an incremental cycle ergometer test in students [15] and a Yo-Yo intermittent recovery test in young soccer players [12]. While there is a good theoretical basis for performance differences due to hormonal control of glucose metabolism [13], results from laboratory and field studies yield heterogenic findings. Additionally, no differences in endurance performance and RPE were found for the field test (i.e., 3.000-m run). One possible explanation for the results of the 3.000-m run is that the self-selected pacing is a crucial factor for maximum performance in the 3.000-m run [30]. This is supported by the reported mean RPE which did not reach the range of exhaustion criteria (RPE  > 16) in the 3.000-m run, while exhaustion criteria were reached (mean max lactate  > 9 mmol/l) [24] in the incremental treadmill test.

Furthermore, no evidence for a daytime variation in any physiological parameter was found in our study. Contrasting, previous studies found higher blood lactate levels for various exercises [4, 21]. Additionally, one study reported higher blood lactate levels at rest in the morning compared to the afternoon and evening [20], and another study found no differences in blood lactate levels throughout the day [29]. Reasons for the different results between the aforementioned studies and the results of the present study can be found in different test procedure and population. Concerning daytime variations of HR during endurance exercise, the overall results seem to be inconsistent [30]. While some studies reported evidence for the presence of daytime variation in HR [16–18], Chtourou and Souissi described equivocal results for daytime variation of HR in their recent review [30].

Overall, our hypotheses that daytime variations are present in endurance performance and related physiological parameters of youth soccer players could not be confirmed by this study. While circadian rhythms are considered an important factor related to physical performance and physiological parameters in competitive sports, the importance of circadian rhythms for aerobic endurance performance remains unclear.

## Limitations

Some limitations must be acknowledged concerning this study. First, the use of only two times of the day (i.e., morning and evening) might not be sufficient because the time window for optimal performance differs for each individuum [2]. However, the choice of the selected times of the day in our study did incorporate the optimum time of day for soccer players’ performance between 04:00 p.m. and 08:00 p.m. [6] to compensate for this shortcoming. Secondly, the RPE used in the 3.000-m run has not been used in the incremental treadmill test, while blood lactate testing has only been performed during the incremental treadmill test and not after the 3.000-m run and therefore limits the interpretation concerning exhaustion criteria. Other important factors might be that this study did not control for sleeping patterns, sleep duration, naps, and morning or evening type of participants which is known to influence the circadian rhythm [2, 31]. Here, the relation between the chronotype and the performance of athletes at certain daytimes is particularly interesting but evidence in the literature is heterogenic [32, 33]. Finally, a larger sample size would have reduced the beta error and would lead to more robust results.

Future studies should address these shortcomings by adding physiological parameters to control for exhaustion criteria with parameters like blood lactate, HR, and RPE. Additionally, sleep related variables, and chronotype of participants should be considered. This may enable researchers to distinguish between physiological and psychological aspects of aerobic endurance performance and to better determine if and why daytime variations are present for the different outcome parameters. Finally, if studies aim to determine sport-specific (i.e., soccer) daytime variation, a field test representing the sport-specific requirements seems more appropriate compared to generic endurance tests like the 3.000-m run.

## Supplementary Information

**Additional file 1.** Exercise data of soccer players.

## Data Availability

All data generated or analysed during this study are included in this published article [and its Additional file 1].

## Abbreviations

ES: Effect size (Cohen’s d)
HR: Heart rate
IAT: Individual anaerobic threshold
LT: Individual aerobic threshold
max: Maximal
RPE: Rate of perceived exertion
